# A Pilot Study to Assess Opportunistic Use of CT-Scan for Osteoporosis Screening in Chronic Pancreatitis

**DOI:** 10.3389/fphys.2022.866945

**Published:** 2022-05-31

**Authors:** Julia McNabb-Baltar, Hanisha R. Manickavasagan, Darwin L. Conwell, Andrew Lu, Dhiraj Yadav, Philip A. Hart, Luis F. Lara, Zobeida Cruz-Monserrate, Steven Ing, Alice Hinton, Thomas A. Mace, David Bradley, Zarine K. Shah

**Affiliations:** ^1^ Brigham and Women’s Hospital, Harvard Medical School, Division of Gastroenterology, Hepatology, and Endoscopy, Boston, MA, United States; ^2^ The Ohio State University Wexner Medical Center, Division of Gastroenterology, Hepatology, and Nutrition, Columbus, OH, United States; ^3^ The Ohio State University Wexner Medical Center, Department of Radiology, Columbus, OH, United States; ^4^ University of Pittsburgh Medical Center, Division of GI, Hepatology and Nutrition, Columbus, OH, United States; ^5^ The Ohio State University Wexner Medical Center, Division of Endocrinology, Diabetes and Metabolism, Columbus, OH, United States; ^6^ Division of Biostatistics, College of Public Health, The Ohio State University, Columbus, OH, United States

**Keywords:** chronic pancreatitis (CP), osteoporosis, DXA (dual-energy X-ray absorptiometry), opportunistic screening, CT scan

## Abstract

**Objectives:** CT scans are commonly performed in patients with chronic pancreatitis (CP). Osteopathy and fractures are recognized in CP but no osteoporosis screening guidelines are recommended. “Opportunistic” CT scan-derived bone density thresholds are assessed for identifying osteoporosis in CP.

**Methods:** Retrospective pilot cohort study. CP subjects who had CT scans and dual-energy x-ray absorptiometry (DXA) within 1 year were included. CT-derived bone density was measured at the L1 level. Pearson’s correlation was performed between age and CT-derived bone density in Hounsfield unit (HU). Univariate analysis using HU to identify osteoporosis was performed at various thresholds of bone density. The discriminatory ability of the model was evaluated with the area under the receiver operating characteristic (ROC) curve (AUC). Several HU thresholds were tested.

**Results:** Twenty-seven CP subjects were included, of whom 11 had normal bone density, 12 osteopenia, and four osteoporosis on DXA. The mean age was 59.9 years (SD 13.0). There was a negative correlation of age with HU (r = −0.519, *p* = 0.006). CT-derived bone density predicted DXA-based osteoporosis in the univariable analysis (Odds Ratio (OR) = 0.97 95% Confidence Interval (CI) 0.94–1.00, *p* = 0.03). HU thresholds were tested. A threshold of 106 HU maximized the accuracy (AUC of 0.870).

**Conclusions:** CT scan may be repurposed for “opportunistic” screening to rule out osteoporosis in CP. A larger study is warranted to confirm these results.

## Introduction

Chronic pancreatitis (CP) predisposes to metabolic bone disease (osteopenia and osteoporosis) and low trauma fracture. The prevalence of osteoporosis or osteopenia in CP is estimated to be 65% (Duggan et al., 2014). We have reported that CP subjects are at high risk for osteoporotic fracture compared to other “high-risk” gastrointestinal conditions for which screening guidelines are in place (Tignor et al., 2010). Yet, only a fraction of CP subjects receive dual-energy X-ray absorptiometry (DXA) imaging testing (Srivoleti et al., 2021). Identification of metabolic bone disease by DXA in CP may lead to interventions that lower fracture risk. However, there are no societal guidelines recommended for osteoporosis screening in CP; thus, insurance carriers do not universally cover costs of DXA “solely” for CP. Therefore, a cost-effective alternative screening method may identify those at risk of fracture (Anderson et al., 2018).

Opportunistic screening for osteoporosis with computerized tomography (CT) scans has been proposed as a no added cost strategy (Pickhardt et al., 2013; Zysset et al., 2015; Jang et al., 2019). Opportunistic use of CT scan is not a substitute for DXA scan but aids in the identification of subjects who are not, otherwise, suspected to be at risk of fracture or osteoporosis. There is grade-A evidence (Pickhardt et al., 2011; Pickhardt et al., 2013; Pickhardt et al., 2015; Schreiber et al., 2015; Zysset et al., 2015; Lee et al., 2016; Gausden et al., 2017; Gerety et al., 2017) that CT opportunistic screening is reproducible and valid compared with DXA to identify osteoporosis (Wright, 2006).

Chronic pancreatitis subjects have numerous CT scans during their lifetime for disease management, which potentially could be repurposed for osteoporosis risk assessment (Mortele et al., 2011). Retrieval of bone density data from CT scans ordered for other indications requires no additional cost, subject time, equipment, or radiation exposure. In addition, data can be obtained retrospectively or prospectively (Wright, 2006; Buckens et al., 2015; Gausden et al., 2017; Lee et al., 2017; Lenchik et al., 2018). Integration of opportunistic CT screening into the evaluation of CP subjects could markedly expand screening efforts in the population and improve our understanding of the burden of bone disease in CP.

The aim of the pilot study was to investigate CT-derived bone density thresholds from a cohort of CP subjects and test their added value for predicting osteoporosis in DXA-derived bone density measurements (lowest axial skeleton T-score). Diagnostic performance of CT-derived bone density (sensitivity, specificity, and accuracy) at different cut points for osteoporosis was calculated to determine its feasibility as an option for opportunistic screening in CP and is compared to thresholds in the medical literature.

## Methods

Subjects with chronic pancreatitis, 18 years and older, followed at the outpatient Pancreas Clinic at The Ohio State University who underwent both a CT [Siemens scanner (SOMATOM Definition Flash, 128 slice CT] and DXA (GE scanner Lunar, Lunar Prodigy or iDXA) scan within 1 year were identified for inclusion into this retrospective pilot study. Using these criteria, we identified 27 subjects with both abdominal CT scans and DXA for analysis. The correlation between CT-derived bone density measured in Hounsfield Units (HUs) at the L1 vertebral level vs. DXA T-score was assessed.

Demographics, laboratory, and imaging findings were abstracted, including age, gender, race, smoking, excessive alcohol use (defined as >2 drinks/day male; >1 drink/day female), use of pancreatic enzyme replacement therapy (PERT), diabetes, and vitamin A, D, and E values, when available. Diagnosis of chronic pancreatitis was based on the Cambridge classification (Axon et al., 1984). Study approval was obtained from The Ohio State University Institutional Board Review 2019H0353.

**FIGURE 1:**
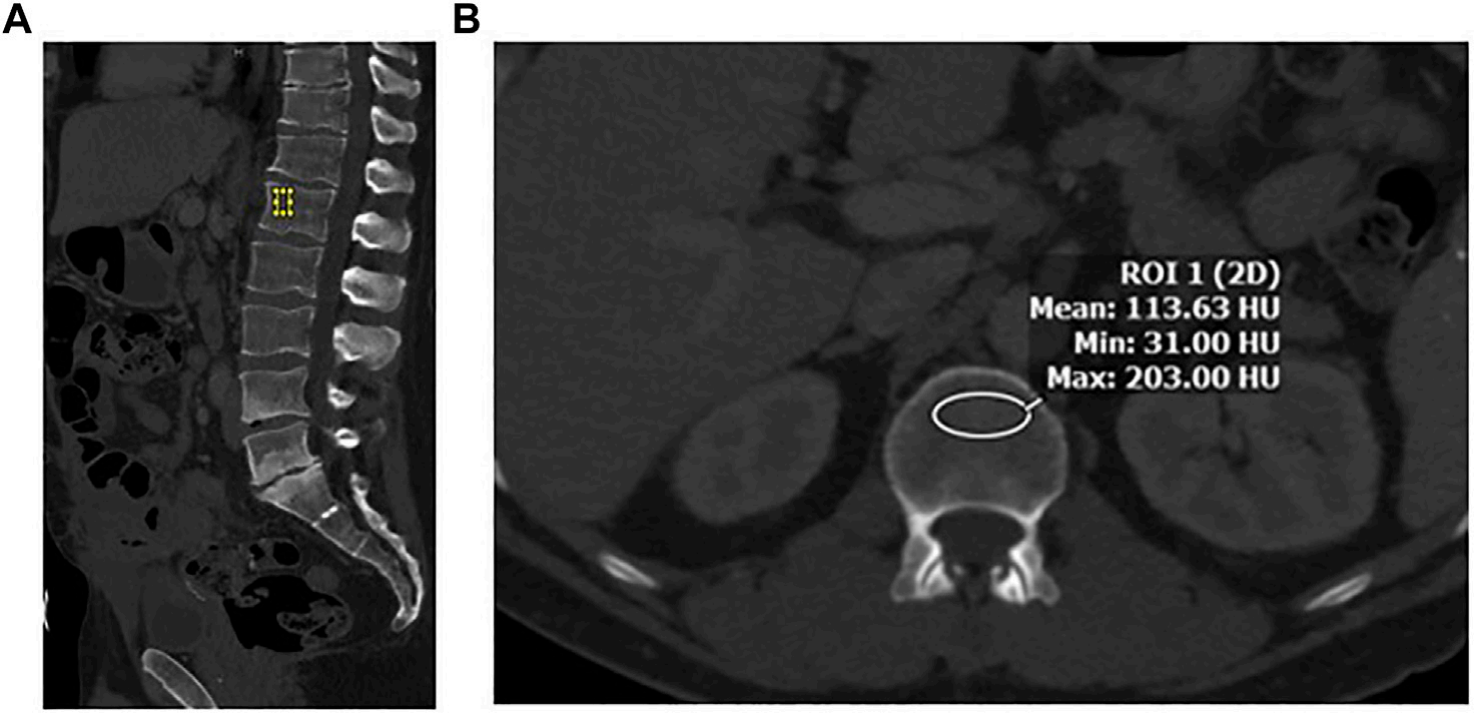
**(A)** Sagittal image in bone windows demonstrating placement of an elliptical region of interest at L1 along the anterior aspect of the vertebral body. **(B)** Axial image at the L1 level along the superior aspect, with an elliptical region of interest to measure CT-derived bone density in Hounsfield Units (HU).

A region of interest (ROI) was drawn manually at the L1 vertebral body. It is the first non–rib-bearing vertebra and can be seen on both abdominal and thoracic CT scans. The L1 vertebra avoids most degenerative processes, and HU thresholds have been determined for L1 with excellent interobserver agreement. The HU value was determined by drawing an ellipse as large as possible on the axial and the midsagittal reconstruction in the anterior two-thirds of the vertebral body (Figure 1). This avoids venous channels in the posterior vertebra and should only include the trabecular bone. Additional images were assessed for occult vertebral fracture. The average of the mean HU values from the ROIs was recorded. Care was taken to note the model and voltage of the CT image and standardization of images and reviewed by a single abdominal radiologist (ZS).

DXA scan results were interpreted by a certified clinical densitometrist (CCD) as per The International Society for Clinical Densitometry (ISCD) guidelines and were recorded for comparison. Osteoporosis, osteopenia, and normal bone density were defined according to DXA results (Zysset et al., 2015).

Statistical Methods: Statistical analysis was conducted using SAS, version 9.4 (SAS Institute, North Carolina, US). The means and standard deviation (SD) were generated for continuously coded variables, whereas frequencies and proportions were generated for categorical variables. The association with CT-derived bone densities at L1 was evaluated with Pearson’s correlations for age. A univariable logistic regression model was fit for osteoporosis to evaluate the contribution of L1 density. The discriminatory ability of the model was evaluated with the area under the receiver operating characteristic (ROC) curve (AUC), and the maximum of the sum of sensitivity and specificity was used to determine the optimal L1 density threshold for distinguishing between normal/osteopenia and osteoporosis. Sensitivity, specificity, positive predictive value (PPV), and negative predictive value (NPV) were calculated using different thresholds to indicate osteoporosis, including a CT derived bone density in HU threshold ≤110, ≤135, ≤160, all derived from previously published literature and the threshold derived from the ROC curve maximizing the sum of sensitivity and specificity in our dataset. (Wright, 2006; Pickhardt et al., 2011; Pickhardt et al., 2013; Pickhardt et al., 2015; Schreiber et al., 2015; Zysset et al., 2015; Lee et al., 2016; Gausden et al., 2017; Gerety et al., 2017; Jang et al., 2019). All tests were two-sided with a statistical significance set at *p* < 0.05.

## Results

### Demographics and Study Population

**FIGURE 2:**
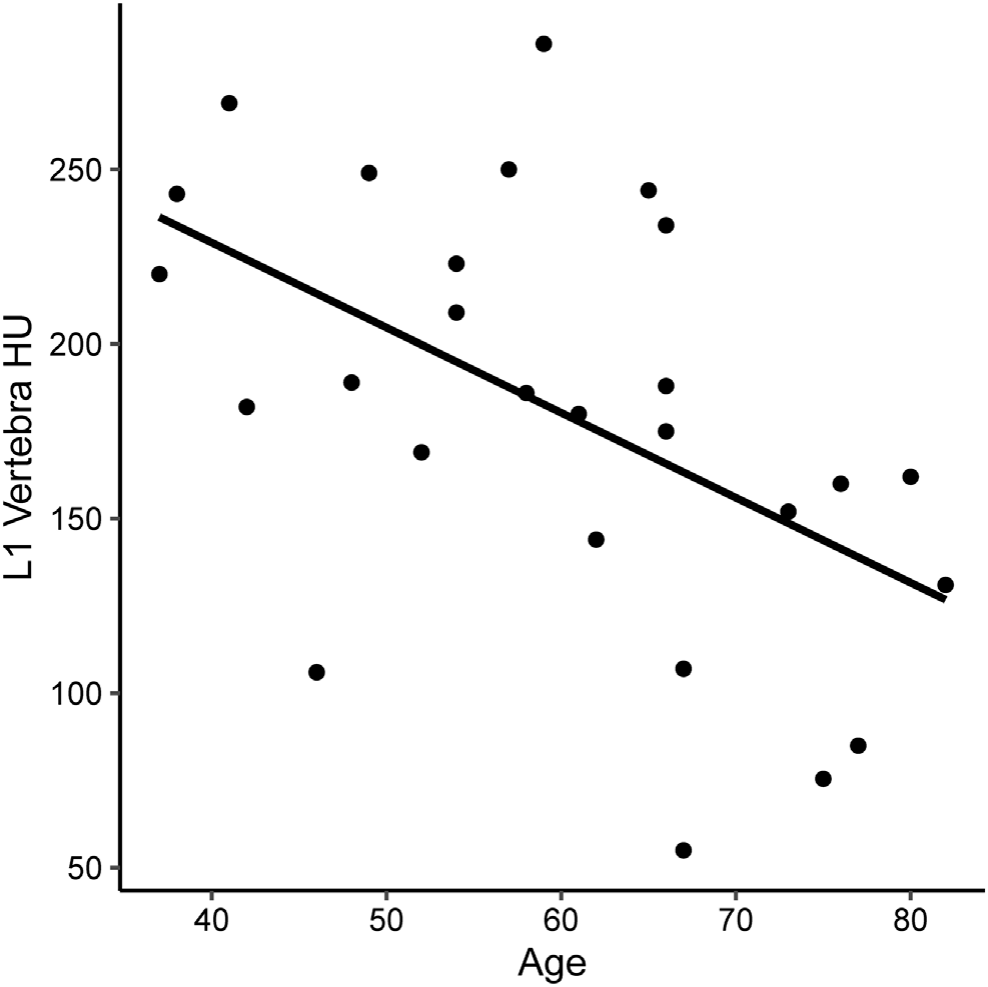
Scatterplot of CT-derived bone density [measures in Hounsfield Units (HU) at the L1 vertebra] in subjects with CP.

A total of 27 subjects were included in this study, of whom 11 had normal bone density, 12 osteopenia, and four osteoporosis on DXA. Table 1 shows the summary demographics of the study population. The mean age was 59.9 years (SD 13.0). Overall, seven subjects were female (25.9%), 2/11 with normal bone density (18.2%), 3/12 with osteopenia (25.0%) and 2/4 with osteoporosis (50.0%). The majority of the study population were white (81.5%), smoking (70.4%), had diabetes (77.8%), and (88.9%) were on PERT, including all the subjects with osteoporosis. The mean levels of fat-soluble vitamins were also recorded and were not statistically significantly different according to the bone health cohorts. Figure 2 shows the correlation and scatterplot of age- and CT scan–derived L1 HU density. Age (r = −0.519, *p* = 0.006) was associated with L1 density in a moderate negative linear relationship.

**TABLE 1:** Summary of the population of CP subjects with both CT and DXA scans and within each of the three cohorts: normal, osteopenia, and osteoporosis.

	Overall (*n* = 27)		Normal (*n* = 11)		Osteopenia (*n* = 12)		Osteoporosis (*n* = 4)	
Age (*mean, SD*)	59.9	13.0	62.7	11.8	56.8	14.4	61.5	12.5
Female (*n, %*)	7	25.9	2	18.2	3	25.0	2	50.0
White race (*n, %*)	22	81.5	10	90.9	9	75.0	3	75.0
Smoking (*n, %*)	19	70.4	8	72.7	7	58.3	4	100.0
Excessive alcohol use (*n, %*)	11	40.7	4	36.4	6	50.0	1	25.0
PERT use (*n, %*)	24	88.9	10	90.9	10	83.3	4	100.0
Diabetes	21	77.8	10	90.9	8	66.7	3	75.0
Vitamin D (*mean, SD*)	28.5	12.6	27.2	12.5	27.5	12.6	35.3	13.9
Vitamin A (*mean, SD*)	42.9	23.3	39.5	20.5	41.9	18.0	55.5	43.0
Vitamin E (*mean, SD*)	7.9	3.7	7.9	4.5	7.8	2.8	8.5	4.9

PERT, pancreatic enzyme replacement therapy.

### CT-Derived Bone Density Compared to Dual-Energy X-Ray Absorptiometry

**FIGURE 3:**
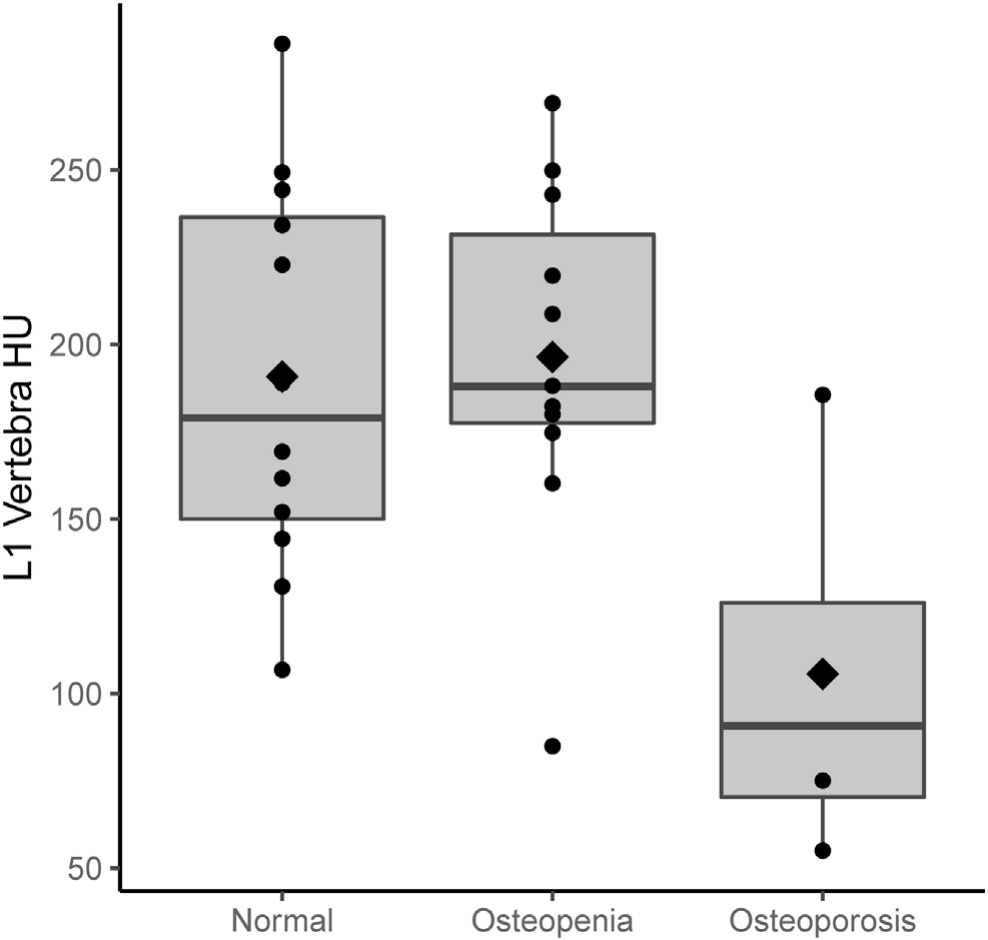
Boxplots of L1 density (HU) by DXA classification; diamonds indicate the mean HU.

**FIGURE 4:**
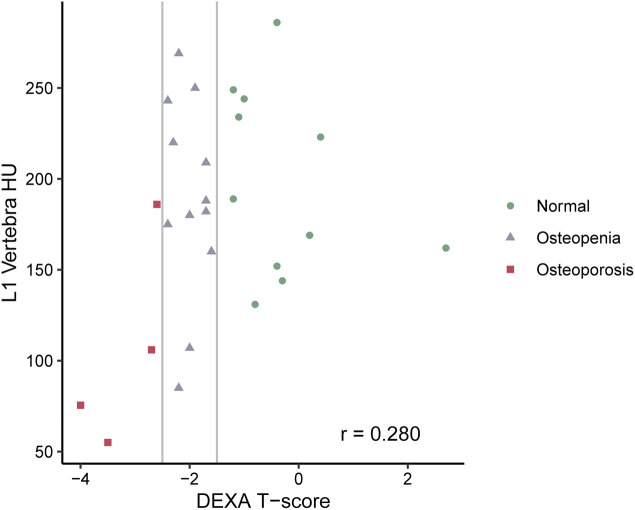
Scatterplot of L1 density (HU) by DXA classification.

**FIGURE 5:**
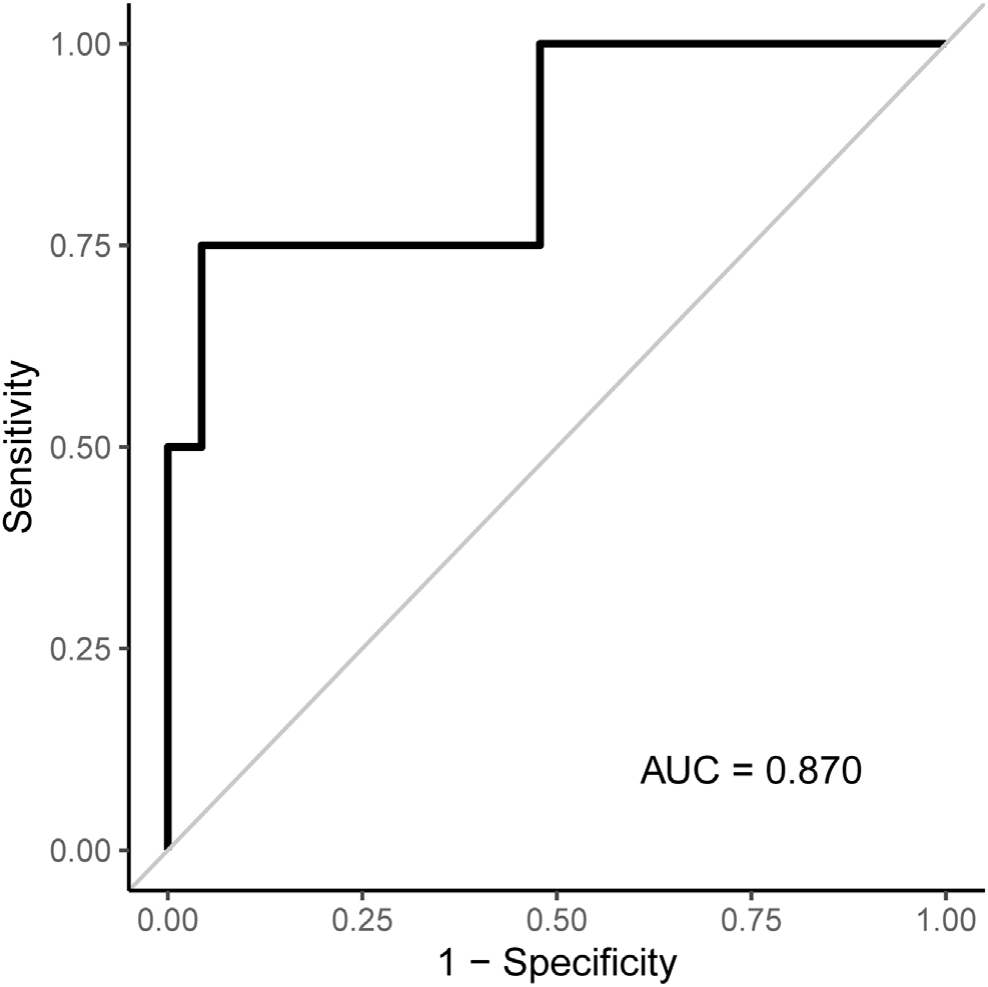
ROC curve associated with the logistic regression model for osteoporosis shown in Table 2. An L1 density threshold of ≤106 to indicate osteoporosis maximizes the sum of sensitivity and specificity within this data.

Figures 3, 4 show the CT-derived bone density within each bone health cohort. A univariable logistic regression model assessing the association between HU and osteoporosis in CP was performed. For every one unit of HU rise, the odds of osteoporosis decreases by 0.03 Odds Ratio (OR) = 0.97 95% Confidence Interval (CI) 0.94–1.0, *p* = 0.03. Figure 5 shows the ROC curve (AUC of 0.87), where a threshold of 106 HU maximizes the sum of sensitivity and specificity within this data.

### Evaluation of CT-Derived Bone Density Thresholds for Osteoporosis

Sensitivity, specificity, PPV, and NPV were assessed according to various thresholds to indicate osteoporosis when applied to our dataset (Table 2). Based on this, a threshold of 106 HU maximizes the sum of sensitivity and specificity. Values at or below 106 classify subjects as having osteoporosis and values >106 as not having osteoporosis. A highly accurate threshold to rule in osteoporosis is difficult to identify with this pilot data as only four subjects had osteoporosis. The four subjects with osteoporosis had L1 densities of 55, 75.7, 106, and 186.

**TABLE 2:** Sensitivity, specificity, and positive and negative predictive values of various L1 density thresholds indicate osteoporosis when applied to our dataset. The 106 and 130 thresholds were derived from the OSU data, while the other thresholds are found in the literature.

Threshold	Sensitivity (%)	Specificity (%)	PPV (%)	NPV (%)
≤106	75	96	75	96
≤110	75	91	60	95
≤130	75	91	60	95
≤135	75	90	50	95
≤160	75	74	33	94

## Discussion

To our knowledge, this is the first report of CT-derived bone density screening in osteoporosis in CP. In this pilot study, we have found that CT has the potential to be repurposed as a screening tool in the setting of CP. We assessed multiple thresholds described in the literature and found that a threshold of 106 HU maximized the sum of sensitivity and specificity. This value is similar to a threshold of 110 which has been advanced as a cut point conferring high specificity to rule in osteoporosis (Pickhardt et al., 2013). Since this dataset contained identical sensitivity values at each threshold, we do not arrive at a cut point to rule out osteoporosis. A larger study is needed to validate the findings.

People with CP have multiple risk factors for osteopathy, including vitamin D malabsorption, malnutrition, exocrine pancreatic insufficiency, smoking, alcohol abuse, and chronic inflammation, and half are female, all of which contribute to bone mineral density loss and fractures (Duggan et al., 2014; Hart et al., 2021). In addition, several reports from our research group have described fractures and osteopathy in CP subjects (Tignor et al., 2010; Munigala et al., 2016; Hart et al., 2021). Since DXA screening for osteoporosis is not universally covered by insurance, an alternative strategy is needed to identify CP subjects at risk for fracture. Opportunistic CT screening may provide a viable, no-cost alternative.

More than 30 million CT scans were performed in the US in 2013, most of which contain information that can be repurposed (Mettler, 2019). In this context, a low or no-cost alternative screening method would be a major advancement in the field of pancreatology. In essence, CT scans ordered for other indications such as trauma and abdominal pain have been repurposed to screen for osteoporosis by measuring linear X-ray attenuation coefficient in Hounsfield Units (HUs). X-ray attenuation is proportional to the atomic mass and atom density of the tissue subjected to irradiation. For bone, HU is proportional to the mineral density. HU is presented on a grayscale for visualization and is easily determined numerically for any planar region of interest (ROI) using radiology software in routine clinical use. CT-derived HU measurements have been shown to strongly correlate with bone mineral density in the general population, and thresholds have been derived to rule in or rule out osteoporosis. A threshold of <110 HU indicates a risk for osteoporosis (Pickhardt et al., 2013). Our pilot data are in-line with prior data on a nonCP population.

Opportunistic CT screening is carried out at the L1 vertebrae. This vertebra is the optimal site to draw an ROI and screen for osteoporosis, especially since this level is in the field of view on both thoracic and abdominal CT scans**.** Population-based, age-related bone density measured at L1 is fairly constant and predictable. Also, postmenopausal women and men have similar trabecular L1 attenuation values (Jang et al., 2019). Standardized protocols and methods have been established, including measurements of HU at a standard 120 kilovolts (kV) (the most common CT scan setting) (Garner et al., 2017). There is no significant difference between L1 HU measurement for scans performed without or with IV contrast material (Pickhardt et al., 2013; Pickhardt et al., 2016; Jang et al., 2019) for middle-aged subjects or for the elderly. Variations in HU at L1 without and with IV contrast have been reported, but the clinical impact of this difference is not likely to be significant when utilizing CT as an opportunistic screening tool for osteoporosis in high-risk subjects such as those with CP. (Wright, 2006; Pickhardt et al., 2016).

We report that HU can be used to screen for osteoporosis in CP with a high correlation to T-scores on DXA, which corroborates previously published literature in other patient populations (Wright, 2006). CP subjects may receive multiple CT scans during the course of their disease, either to assess complications of CP or as a screening tool for early detection of pancreatic cancer. These CT scans obtained as standard of care can be used as an opportunistic screening tool to look for bone loss, which would put these subjects at increased risk for fractures. This can guide clinicians to obtain DXA scans for diagnosis and allow timely treatment interventions to reduce further bone loss.

In our study, population age was moderately inversely correlated with L1 HU, which provided biological plausibility to our findings. Increased age has been associated with decreased bone density (Barkaoui et al., 2017). Multiple factors can play a role in this change, including alterations in the dynamics of bone cells, which can result in alterations in the bone formation and resorption process, changes in the bone architecture, and disparities in the concentration of deposited mineral, leading to hypo-mineralized areas. Some changes in the calcium and phosphate regulation also change with age including increases in parathyroid hormones and decrease in the vitamin D3 active metabolites. Finally, a decrease in physical activity and dietary inadequacies will lead to bone loss (Kiebzak, 1991).

The major limitation of this pilot study is the small number of subjects and those with osteoporosis. Given this, identifying a threshold to rule in osteoporosis cannot be confidently performed at this stage. Due to the limited sample size, we were unable to control for confounders in the analysis. Also, since the reported HU thresholds in the literature may not be applicable to CP subjects, we tested established thresholds and derived CP-specific thresholds for osteoporosis prediction. Factors that can cause variability in HU measurement include variations in CT manufacturers (coefficient of variation 4.9%), day-to-day fluctuations, administration of contrast (can increase HU by 11 units), CT tube voltage (130 kV has an 18% lower HU value than the standard 120 kV), and patient positioning (prone vs. supine) (Wright, 2006). Since CT scans in our study came from one academic center to account for this minor variability, the manufacturer, voltage, and administration of contrast were recorded and assessed for confounding. As opportunistic CT is a screening and not a diagnostic tool, these known variations in HU have been shown to have little impact (Wright, 2006).

To our knowledge, this is the first report of opportunistic CT screening in CP. This screening method may be especially helpful in the context of chronic pancreatitis where current societal guidelines do not currently recommend DXA testing and insurance carriers do not universally cover the expense. Despite its limitations, this retrospective pilot cohort study adds important evidence on expanding the use of opportunistic CT screening for osteoporosis in chronic pancreatitis. CT-derived bone density is correlated with age and T-scores on DXA. A larger cohort study in CP is needed to validate these findings.

## Data Availability

The raw data supporting the conclusion of this article will be made available by the authors, without undue reservation.
